# Immunohistochemistry Study on Androgen and Estrogen Receptors of Rat Seminal Vesicle Submitted to Simultaneous Alcohol-Nicotine Treatment 

**DOI:** 10.22074/cellj.2016.4574

**Published:** 2016-08-24

**Authors:** Mohsen Basiri, Majid Asadi-Shekaari, Masoud Ezzatabdipour, Arash Sarv Azad, Seyed Noureddin Nematollahimahani

**Affiliations:** 1 Neuroscience Research Center, Neuropharmacology Institute, Kerman University of Medical Sciences, Kerman, Iran; 2 Department of Anatomical Sciences, Afzali Pour Medical School, Kerman University of Medical Sciences, Kerman, Iran; 3 Colorectal Research Center, Iran University of Medical Sciences, Tehran, Iran

**Keywords:** Alcohol, Nicotine, Androgen Receptor, Estrogen Receptor, Seminal Vesicle

## Abstract

**Objective:**

Alcohol consumption is habitually accompanied by the use of other psychoactive substances, mostly tobacco. Nicotine and alcohol affect male accessory reproductive glands function. Most studies have been done on pathologic features of prostate, but there has been no systematic study on the seminal vesicle. Therefore, the aim of current study was to investigate the distribution of androgen receptor (AR) and estrogen receptors-beta (ER-β) immune reactivities following long-term treatment of alcohol, nicotine or a combination of both substances.

**Materials and Methods:**

In this experimental study, a total of 40 adult Wistar rats, nine weeks of age, were used. Animals were randomly divided into four groups, including: i. Control group receiving normal saline 0.09%, ii. Ethanol group receiving ethanol 20% (2 ml/kg, via gavage), iii. Nicotine group receiving nicotine (0.1 mg/kg, subcutaneous injection), and iv. Ethanol-nicotine group receiving simultaneous ethanol 20% (2 ml/kg) and nicotine (0.1 mg/kg) treatment. All treatment lasted for eight weeks. Prior to intracardiac perfusion, blood sample was collected from left ventricle. The seminal vesicles were isolated and processed for paraffin blocking. The sample tissues were then studied for distribution of AR and ER-β immunereactivities using immunohistochemical (IHC) staining method. One way analysis of variance (ANOVA) and Tukey’s test were performed for data analysis. A value of P<0.05 was considered significant.

**Results:**

Our results revealed that the lowest mean number of positive cells belonged to the animals of ethanol-nicotine group that was followed by the ethanol, nicotine, and control groups, respectively. However, there was no significant difference regarding serum testosterone level among experimental groups.

**Conclusion:**

It was concluded that combination of both ethanol and nicotine may be a crucial factor in the expression levels of AR and ER-β.

## Introduction

The seminal vesicles are composed of glandular epithelium secreting most (70%) of the seminal fluid. The normal function of the seminal vesicle is important for fertility. Seminal vesicle secretion has fundamental effect on sperm mobility, immune protection and the stability of sperm chromatin. Androgens, as main hormones, play an important role in proliferation, differentiation and function of accessory sex glands in particular seminal vesicle (1,2). Testosterone and estrogen have significant roles in the function of the male reproductive system. High concentration of estrogen has been reported in rete testis fluid; in addition, estrogen receptors have been detected in almost entire parts of reproductive tracts, including seminal vesicle (3,4). It was shown the role of androgen receptors (ARs) in seminal vesicle development. In fact, these receptors induce epithelial cell mitosis, cell morphogenesis as well as secretory modulation of specific proteins. The biological actions of androgens are mediated by interaction with specific intracellular receptors that modulate gene expression by binding to nuclear chromatin (5). Testosterone and dihydrotestosterone (DHT) are the main circulating androgens (6). Alcohol has detrimental effects on the male reproductive system and causes general disturbance in this system (7). Ethanol may either directly change testosterone production in the gonads, or indirectly alter the hypothalamic-pituitary-gonadal axis (HPG axis) (8). Some studies have also verified the following common side effects of chronic alcoholism: testicular lesions, reduced testosterone level and reduction in weight of the accessory sex glands (9,10). 

Cigarette smoking is quite common in the general population, but our knowledge of its effect on seminal vesicle function is still very limited. The main alkaloid of tobacco is nicotine that is responsible for harmful effects in smokers. Nicotine also alters the equilibrium in the male reproductive system (11). Cigarette smoking is associated with a significant decrease in total motile sperm count (TMSC) (12). Chronic nicotine treatment in the rat animal model causes seminal vesicle atrophy (13). Epidemiological study has shown that around 90% of alcoholics also smoke cigarettes (14). Most studies have focused on testes and sperm cells in different animal models, but there are no systematic studies on the effect of alcohol, nicotine and the combination of both on accessory male genital glands, particularly on structure of seminal vesicle. Therefore, the aim of the current study was to investigate the effects of long-term ethanol and/or nicotine administration on distribution of AR and estrogen receptors-beta (ER-β) immunoreactivities in adult rat. 

## Materials and Methods

This experimental study was performed based on animal experimental protocols of Kerman University of Medical Sciences. The protocol was approved by the Ethics Committee (EC/VCR/ 89-4) of Kerman University of Medical Sciences. 

### Animals and tissue preparation

A total of 40 male rats were divided into four groups (n=10) as follows: i. Control group receiving normal saline 0.09%, ii. Ethanol group receiving ethanol 20% (2 ml/kg, via gavage), iii. Nicotine group receiving nicotine (0.1 mg/kg, subcutaneous injection), and iv. Nicotine-ethanol group receiving simultaneous ethanol 20% (2 ml/kg) and nicotine (0.1 mg/kg) treatment. After eight weeks of treatment, the animals were anaesthetized by 400 mg/ kg chloral hydrate (Mina Tajhiz Aria, Iran), while after the animals were transcardially perfused with 10% formaldehyde (Arta Razi Teb Co., Iran) in 0.1 M phosphate buffer saline (PBS, pH=7.4, SigmaAldrich, USA), the sample blood was collected. The seminal vesicles were isolated and processed for immunohistological studies. 

### Hormone concentrations in serum

At the end of the 56-day treatment, blood samples were collected from all rats in the control and treatment groups, and the serum testosterone level was then determined by radioimmunoassay using Coat-A-Count total testosterone direct kit (Diagnostic Products Corporation, USA). The serum hormone concentrations were expressed in ng⁄ml. 

### Light microscopy

After collecting the samples of the seminal vesicle from all animals of the control and treatment groups, they were proceed for paraffin embedding, cut into 3-µm-thick sections and submitted to immunohistochemical (IHC) staining method. 

### Immunolocalization of androgen receptor and β-estrogen receptor

The sections were deparaffinized in xylene, hydrated through a graded alcohols series and rinsed in tap water. Antigens were retrieved by boiling the sections in a 10-mm citrate buffer, pH=6.0, three times for 5 minutes in a microwave oven. The cooled sections were incubated in 0.3% H_2_O_2_(Kimia Pars, Iran) for 15 minutes to block endogenous peroxidase. Non-specific binding was blocked by incubating the sections in blocking solution for one hour at the room temperature. Rabbit primaryantibody N-20 (sc-816, Santa Cruz Biotechnology, Inc., USA) for the AR and mouse primary antibody H-150 (sc-8974, Santa Cruz Biotechnology, Inc., USA) for the ER-β were first diluted in 1% bovine serum albumin (BSA, 1:50, Sigma-Aldrich, USA) and then incubated with the sections overnight at 4˚C. Afterwards, the sections were washed for 15 minutes with Tris-buffered saline (TBS)-T (Sigma-Aldrich, USA) and incubated with horseradish peroxidase (HRP)-conjugated anti-rabbit and antimouse antibodies (sc-3837 and sc-3697; Santa Cruz Biotechnology, Inc., USA) at a dilution of 1:100 for 2 hours at room temperature. After washing in TBS-T, peroxidase activity was detected with 3, 3-diaminobenzidine (DAB, Abcam, UK) as the chromogenic substrate. Sections were lightly counterstained with haematoxylin (Sigma-Aldrich, USA), dehydrated in an increasing ethanol series and xylene, and then mounted in Entellan (Merck, Germany). 

### Counting of immunolabelled androgen receptor and β-estrogen receptor

The immunolocalization of AR and ER-β was performed for all animals in experimental groups. Five microscopic fields per animal were measured with an objective lens (magnification ×40). The total number of ARand ER-β-positive cells were expressed as the percentage of these cells, and if they were quantified based on the area of positive immunostaining, they would expressed as a percentage of the total area examined. 

### Statistical analysis

The serum testosterone level as well as the percentage of ARand β-ER-β-positive cells were compared between groups and analyzed statistically by means of analysis of variance (ANOVA) and Tukey’s range test, with the level of significance set at 1 and 5%. 

## Results

### Hormone concentrations in serum

The average values of the serum testosterone levels did not differ significantly among groups.

**Table 1:** Comparison of testosterone levels among different groups


Groups	Testosterone level (ng/ml)

Control	10.85 ± 0.8
Ethanol	11.18 ± 0.7
Nicotine	10.40 ± 0.7
Ethanol-nicotine	10.90 ± 0.9


### Immunolocalization of androgen receptor 

**Fig.1:**
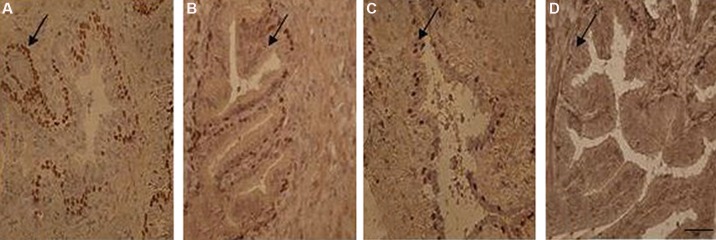
Photomicrograph of immunohistochemical study on rat seminal vesicles belonging to experimental groups. A. AR of control, B. Ethanol, C. Nicotine, and D. Ethanol-nicotine groups, respectively. Arrow points to AR-positive cells (scale bar: 50 µm). AR; Androgen receptor.

The secretory epithelium showed columnar cells with dense brown positive androgen receptor in nuclei, in control group (Fig.1A). The number of positive androgen receptor in nuclei of the secretory epithelium in the ethanol group reduced comparing to control group (Fig.1B). There was also a reduction in the number and density of the secretory epithelial cells in nicotine group comparing to control group, although this was less marked than in the ethanol group (Fig.1C). Simultaneous nicotine and ethanol administration resulted in atrophic epithelial cells with the lowest number of positive androgen receptor cells (Fig.1D).

### Immunolocalization of β-estrogen receptor

**Fig.2:**
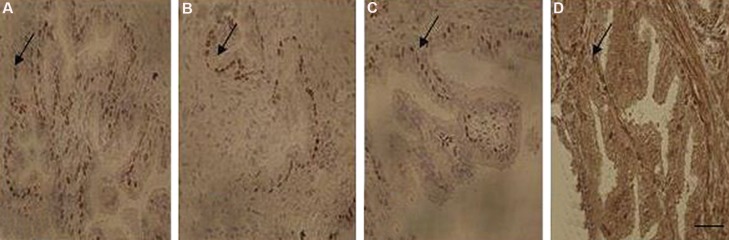
Photomicrograph of immunohistochemical study on rat seminal vesicles belonging to experimental groups. A. ER-β of control, B. Ethanol, C. Nicotine, and D. Ethanol-nicotine groups, respectively. Arrow points to ER-β-positive cells (scale bar: 50 µm). ER-β; Estrogen receptor-β.

The secretory epithelium of rat’s vesicle seminal demonstrated columnar cells with dense brown positive β-estrogen in nuclei in control group (Fig.2A). The reduction of β-estrogen receptor in nuclei of the secretory epithelium was seen in the ethanol group comparing to control (Fig.2B). There was also a reduction in the number and density of the secretory epithelial cells in nicotine group comparing to control, although this was less marked than in the ethanol group (Fig.2C). Simultaneous nicotine and ethanol administration resulted in atrophic epithelial cells with the lowest number of positive β-estrogen cells (Fig.2D).

**Fig.3:**
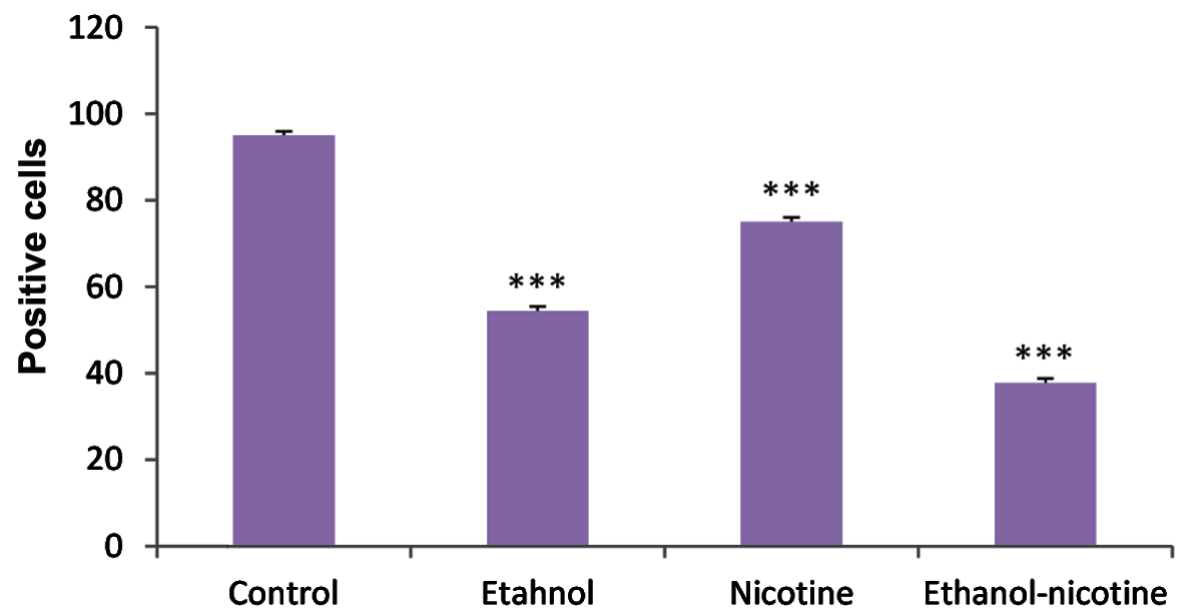
The number of AR-positive cells in experimental groups. ***; A significant difference between control and treatment groups (P<0.000) and AR; Androgen receptor

The secretory epithelial cells contained 94.9 ± 0.21%, 54.4 ± 0.28%, 75.0 ± 0.22% and 37.7 ± 0.24% of the AR immunoreactivity in control, ethanol, nicotine and ethanol-nicotine groups, respectively. It means that simultaneous ethanol and nicotine administration resulted in atrophy of epithelium (Fig.3).

**Fig.4:**
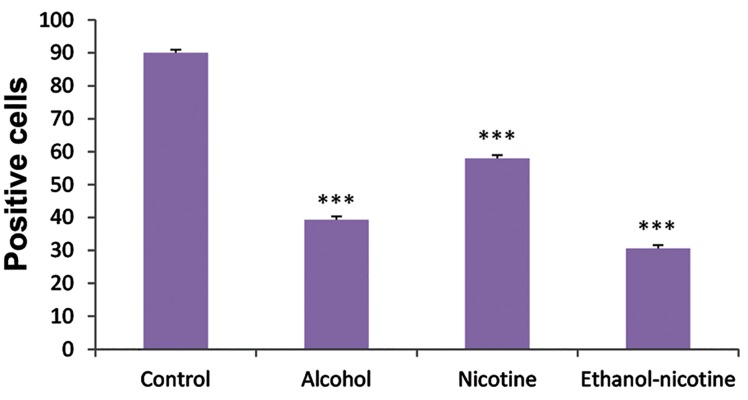
The number of ER-β-positive cells in experimental groups. ***; A significant difference between control and treatment groups (P<0.000) and ER-β; Estrogen receptor-β.

The ER-β immunoreactivity was observed in 90.1 ± 0.17%, 39.3 ± 0.54%, 57.9 ± 0.34% and 30.6 ± 0.30% of the total measured cells in control, ethanol, nicotine and in ethanol-nicotine groups, respectively (Fig.4).

## Discussion

It has been reported that simultaneous nicotinealcohol use has detrimental effects on various organic systems including the genital system (15). However, most studies have focused on the morphology and pathology of prostate diseases. To our knowledge, there was no systematic investigation regarding the effects of concurrent alcohol and nicotine use on distribution of AR and ER-β immunoreactivities. 

Our previous morphological study in rats has showed atrophied epithelium of seminal vesicle, especially in the ethanol group as compared to the control group (16). In the current IHC analysis, we detected a significant reduction in expression levels of AR and ER-β in the ethanol-nicotine group as compared to control and other treatment groups. As a result, alcohol and nicotine use may lead to impairment of secretory function in seminal vesicles. This association is related to pathogenesis of glandular dysfunction (17). According to Meikle et al. (18), nicotine and its metabolite, cotinine, are the inhibitors of 3-hydroxysteroide dehydrogenase, an important enzyme in testosterone and DHT metabolism, which can alter the androgenic action on seminal vesicle. The seminal vesicle is a hormonedependent male accessory sex gland (19). 

In the male reproductive system, decreased fertility is pointed out as a consequence of the action of nicotine on the gonads in long-term alcohol or/ and nicotine treatment (15). Therefore, the current study established common characteristics of longterm alcohol and nicotine use with pathogenesis of glandular dysfunction. However, we detected no significance difference regarding the serum testosterone level among the treatment groups that may indicate the low doses of nicotine and/ or alcohol used for the treatment groups. Besides, the time frame of this experiment was just eight weeks that may not be adequate time to create noteworthy differences in the testosterone level. 

Alcohol and its metabolites, especially acetaldehyde, lead to general disturbance in various organ systems, like male reproductive system, resulting in hypogonadism (7). Experimental and clinical studies have indicated that continued ingestion of ethanol contributes to testicular luteinizing hormone receptors depletion, gonadotropin responsiveness, and disturbances in HPG axis, which provoke damage in male sex organogenesis that leads to abnormal secretory activity of the male reproductive tract (20,21). In addition, alcohol acts directly on the male gonads by altering testicular testosterone synthesis (22,23). In a study on rats chronically treated with alcohol, Klassen and Persaud (24) have reported lower folding of the glandular mucosa. Later, Gomes et al. (7) have observed a significant reduction in seminal vesicle weight, atrophy of the secretory epithelium, and partial cell degeneration in rats treated with ethanol for 204 days as compared to the control. Furthermore, after treating adult Wistar rats with sugar cane brandy dissolved in 30 degrees Gay Lussac (v/v) for 60, 120 and 180 days, Martinez et al. (25) have observed marked morphological changes in the secretory epithelium of the seminal vesicle, among which a significant reduction in epithelial cell height, number of secretory vacuoles, and amount of microvilli, in terms of exposure time and alcoholic dosage. In our previous study, examination of seminal vesicles has shown remarkable reduction in fluid secretion in the alcohol-treated group as compared to control group (16). 

Besides, histologic examinations of the testes of chronic alcoholics have demonstrated a marked reduction in seminiferous tubular diameter. This reduction in seminiferous tubular size is a consequence of a reduced number of germ cells within the seminiferous tubules (26). Significant reduction of testicular glutathione levels has been reported as a result of ethanol metabolism in testes (27). In addition, it has been shown that ethanol exposure adversely affects the secretory function of Sertoli cells (28) and disrupts the blood-testis barrier (29). Hence, it seems germ cell maturation was affected by ethanol exposure that may lead to increasing germ cell degeneration, resulting in testicular atrophy and infertility. 

## Conclusion

It can be concluded that ethanol in association with nicotine caused harmful damage to AR and ER-β of rat seminal vesicle. 
